# Cardiac troponin I for predicting right ventricular dysfunction and intermediate risk in patients with normotensive pulmonary embolism

**DOI:** 10.1007/s12471-014-0628-7

**Published:** 2014-12-12

**Authors:** K. Keller, J. Beule, A. Schulz, M. Coldewey, W. Dippold, J. O. Balzer

**Affiliations:** 1 Department of Medicine II, University Medical Center Mainz (Johannes Gutenberg-University Mainz), Langenbeckstr. 1, 55131 Mainz, Germany; 2 Center for Thrombosis and Haemostasis, University Medical Center Mainz (Johannes Gutenberg-University Mainz), Mainz, Germany; 3 Department of Internal Medicine, St. Vincenz and Elisabeth Hospital Mainz (KKM), Mainz, Germany; 4 Department of Radiology and Nuclear Medicine, Catholic Clinic Mainz (KKM), Mainz, Germany; 5 Department of Diagnostic and Interventional Radiology, University Clinic, Johann Wolfgang Goethe-University Frankfurt/Main, Frankfurt am Main, Germany

**Keywords:** Pulmonary embolism, Thrombosis, Venous thromboembolism, Cardiac troponin I, Tachycardia

## Abstract

**Background:**

Right ventricular dysfunction (RVD) and cardiac troponin I (cTnI) are important tools for risk stratification in pulmonary embolism (PE). We investigate the association of RVD and cTnI in normotensive PE patients and calculate a cTnI cut-off level for predicting RVD and submassive PE.

**Methods:**

Clinical, laboratory, radiological and echocardiagraphic data were analysed. Patients were categorised into groups with or without RVD and compared focussing on cTnI. Effectiveness of cTnI for predicting RVD and submassive PE was tested.

**Results:**

One hundred twenty-nine normotensive PE patients, 71 with and 58 without RVD, were included. Patients with RVD were older (75.0 years (61.3/81.0) vs. 66.0 years (57.7/75.1), *P* = 0.019). cTnI (0.06 ng/ml (0.02/0.23) vs. 0.01 ng/ml (0.00/0.03), *P* < 0.0001) and D-dimer values (2.00 mg/l (1.08/4.05) vs. 1.23 mg/l (0.76/2.26), *P* = 0.016) were higher in PE with RVD. cTnI was associated with RVD (OR 3.95; 95 % CI 1.95–8.02, *p* = 0.00014). AUC for cTnI diagnosing RVD was 0.79, and for submassive PE0.87. Cut-off values for cTnI predicting RVD and submassive PE were 0.01 ng/ml, with a negative predictive value of 73 %. cTnI was positively correlated with age, D-dimer and creatinine.

**Conclusions:**

In normotensive PE patients, cTnI is helpful for risk stratification and excluding RVD. cTnI elevation is correlated with increasing age and reduced kidney function.

## Introduction

Acute pulmonary embolism (PE) is potentially life-threatening [1–11]. Mortality from an acute PE event is closely related to the initial haemodynamic status and cardiac adaptation [1, 2, 4, 10, 12–17]. The diagnostic finding of right ventricular dysfunction (RVD) or positive cardiac troponin (cTn) levels in normotensive PE patients appears to alter the patient’s prognosis significantly [1–4, 7–9, 11–14, 18–26].

We aimed to investigate the association of RVD and cardiac troponin I (cTnI) in normotensive PE patients and the potential cTnI for exclusion of RVD. Moreover, a further objective was to investigate parameters that have an impact on elevation of cTnI levels.

## Patients and methods

We performed a retrospective analysis of normotensive patients with confirmed acute PE, who were treated in the Internal Medicine Department of the St. Vincenz and Elisabeth Hospital Mainz, Germany, between May 2006 and June 2011. Patients were identified by searching the hospital information system database (ICD diagnostic code of PE: I26).

## Enrolled subjects

Patients were eligible for our analysis if they had a confirmed acute PE, were treated in the Internal Medicine Department of the hospital, were haemodynamically stable (systolic blood pressure ≥90 mmHg) according to the European Society of Cardiology (ESC) guidelines [4] and American Heart Association (AHA) scientific statement [21], an accurate transthoracic echocardiography (TTE) of acute phase was assessed and patients were at least 18 years of age. Diagnosis of acute PE was confirmed by an identified filling defect in the pulmonary artery system in a computed tomography pulmonary angiogram of the chest (CT) or positive venous ultrasound/phlebography of an extremity consistent with deep vein thrombosis (DVT) in patients with typical symptoms of PE (chest pain or dyspnoea) and a detected positive D-dimer or scintigraphic ventilation-perfusion (V/Q) scan read as high probability for PE. If the diagnosis of PE was not confirmed or the criteria mentioned above were not fulfilled, patients were not included in this analysis. Studies with retrospective analysis of diagnostic standard data do not need an ethics statement in Germany.

## Analysed data

Retrospectively analysed data comprised symptoms, medical history, physical examination, laboratory parameters (cTnI, creatine kinase (CK), creatinine, D-dimer), complications and examination results.

## Definitions

Per definition, cTnI values were elevated if they exceeded 0.1 ng/ml, and D-dimer values if they exceeded 0.110 mg/l. RVD in TTE was defined according to the ESC guidelines [4] and AHA scientific statement [21] as abnormal motion of the interventricular septum, right ventricular (RV) hypokinesis, pressure overload with tricuspid valve insufficiency or RV enlargement (quotient of end-diastolic lateral-septal RV diameter/end-diastolicleft ventricular lateral-septal diameter>0.9, referring to AHA scientific statement [21]).

According to the ESC guidelines [4] and AHA scientific statement [21] the PE patients of our study with RVD or pathological cTnI levels were classified as submassive PE with an intermediate risk and those without both were classified as low-risk PE.

## Study groups

According to TTE results the normotensive PE patients were included in one of the two study groups: the PE group with RVD or the PE group without RVD.

## Statistical analysis

We compared the groups especially focussing on cTnI. Effectiveness of cTnI to predict RVD was tested. Continuous distributed variables were described by median and interquartile range and tested with the Mann–Whitney U-test if they were not normally distributed or by mean, standard deviation (SD) and tested with the Student *t*-test if distribution was normal. Discrete variables were compared with the Chi-square test. A value of *P* < 0.05 was considered to be statistically significant.

Receiver operating characteristic (ROC) curve and Youden index were calculated to test the effectiveness of cTnI to predict RVD as well as cTnI to predict submassive PE. Multivariate regression analysis was performed to test coherence of cTnI, gender, age, CK, creatinine, and D-dimer on RVD. Spearman’s rank correlation was calculated for age, cTnI, CK, creatinine, D-dimer, systolic blood pressure, diastolic blood pressure, heart rate and systolic pulmonary artery pressure (sPAP).

R version 2.14.1 from R Development Core Team (2011) (R Foundation for Statistical Computing, Vienna, Austria) was used for data processing.

## Results

Altogether, 182 patients with acute and confirmed PE were identified in the hospital information system database, but only 129 patients met the criteria of inclusion; 77 women and 52 men with normotensive PE were included in this analysis. The diagnosis of PE was confirmed in 84.4 % by CT,10.1 % by V/Q scan and 5.5 % by DVT diagnosis in ultrasound/phlebography with typical symptoms of PE and positive D-dimer.

**Fig. 1:**
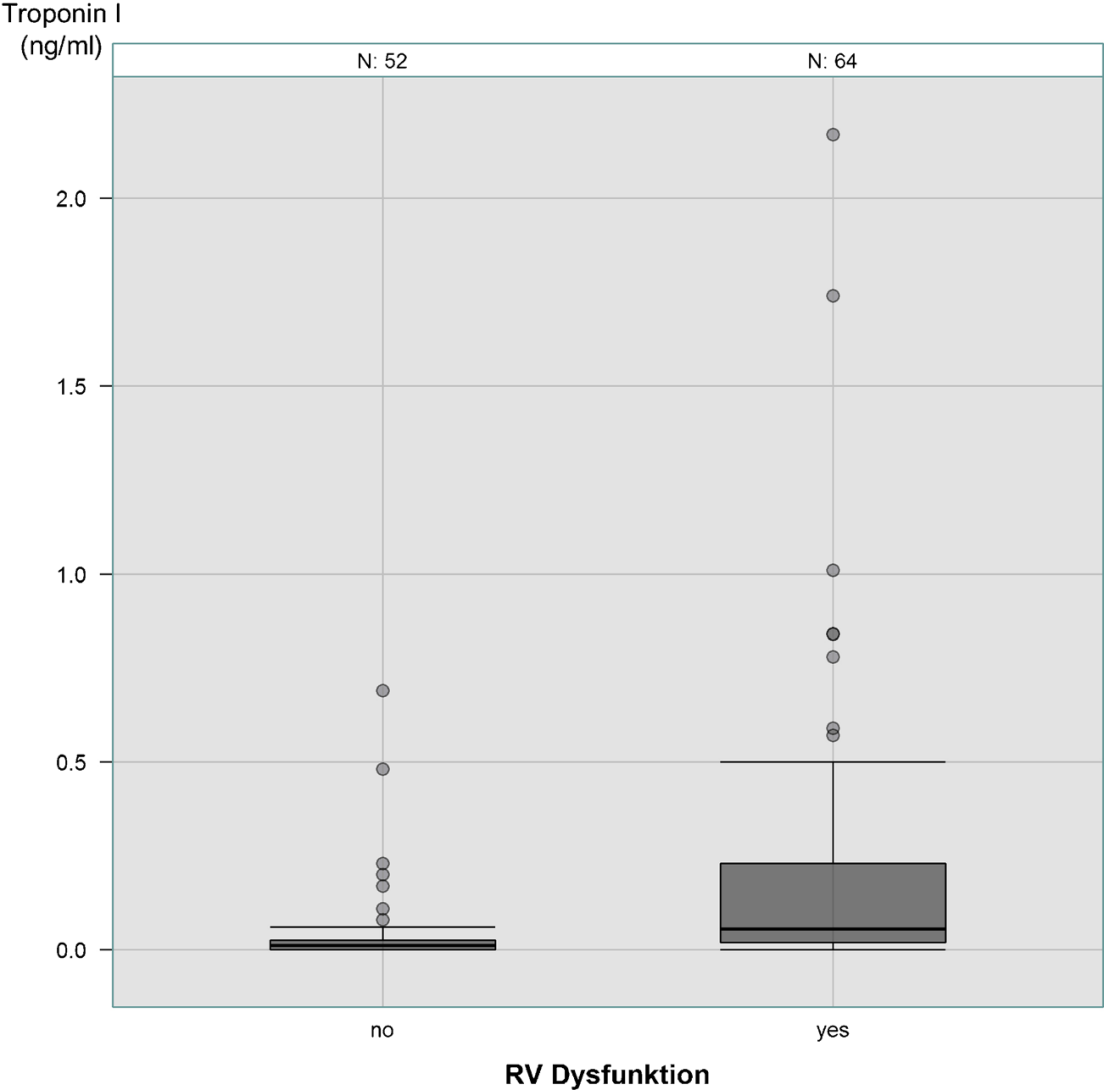
Cardiac troponin I level in PE patients with and without RVD

While 45.0 % of the PE patients did not show RVD in TTE, 55.0 % had RVD criteria. Patients with RVD were older (75.0 years (61.3/81.0) vs. 66.0 years (57.7/75.1), *P* = 0.019), showed higher levels of heart rate (100.0 beats/min (85.3/108.0) vs. 80.5 beats/min (70.0/97.2), *P* < 0.0001), sPAP (43.00 ± 16.06 mmHg vs. 22.79 ± 14.89 mmHg, *P* < 0.0001), cTnI (0.06 ng/ml (0.02/0.23) vs. 0.01 ng/ml (0.00/0.03), *P* < 0.0001) (Fig. 1), D-dimer (2.00 mg/l (1.08/4.05) vs. 1.23 mg/l (0.76/2.26), *P* = 0.016) and creatinine (1.10 mg/dl (0.90/1.38) vs. 1.00 mg/dl (0.80/1.11), *P* = 0.0062), more frequently had a lung infarction with pneumonia (52.1 vs. 31.0 %, *P* = 0.026) and an incomplete or complete right bundle branch block (RBBB) (19.7 vs. 3.5 %, *P* = 0.013) (Table 1). cTnI was not higher in PE patients >65 years than in those ≤65 years (0.03 ng/ml (0.01/0.16) vs. 0.02 (0.00/0.08), *P* = 0.10). In the PE group without RVD, 10.3 % of patients showed positive cTnI values. Of the 129 PE patients, 52 were classified as low-risk PE and 77 as submassive PE (Table 1).

**Table 1:** Characteristics of normotensive PE patients with and without RVD

	PE without RVD (*n* = 58)	PE with RVD (*n* = 77)	*p*-value
Sex (women)	58.6 % (34)	60.6 % (43)	0.97
Age (years)	66.0 (57.7/75.1)	75.0 (61.3/81.0)	**0.019**
Comorbidities			
Surgery or trauma in last 3 months before PE event	24.1 % (14)	14.1 % (10)	0.22
DVT or PE in patient’s history	24.6 % (14)	25.4 % (18)	0.92
DVT	72.4 % (42)	69.0 % (49)	0.82
Lung infarction with pneumonia	31.0 % (18)	52.1 % (37)	**0.026**
In-hospital death	0 % (0)	1.4 % (1)	0.92
Symptoms			
Chest pain	34.5 % (20)	32.4 % (23)	0.95
Dyspnoea	84.5 % (49)	85.9 % (61)	0.98
Haemoptysis	3.4 % (2)	4.2 % (3)	0.82
Syncope or collapse	5.2 % (3)	14.1 % (10)	0.17
Tachycardia	20.7 % (12)	52.1 % (37)	**0.00051**
Physical examination			
Systolic blood pressure (mmHg)	151.4 ± 23.5	143.6 ± 24.8	0.070
Diastolic blood pressure (mmHg)	80.9 ± 20.4	79.5 ± 15.3	0.65
Heart rate (beats/min)	80.5 (70.0/97.2)	100.0 (85.3/108.0)	**< 0.0001**
ECG			
Incomplete or complete RBBB	3.5 % (2)	19.7 % (14)	**0.013**
S1Q3type	3.5 % (2)	14.1 % (10)	0.083
Laboratory			
Cardiac troponin I (ng/ml)	0.01 (0/0.03)	0.06 (0.02/0.23)	**< 0.0001**
Creatine kinase (U/l)	62.0 (44.0/87.0)	65.0 (41.0/105.3)	0.71
Creatinine (mg/dl)	1.00 (0.80/1.11)	1.10 (0.90/1.38)	**0.0062**
D-dimer (mg/l)	1.23 (0.76/2.26)	2.00 (1.08/4.05)	**0.016**
Echocardiography			
Systolic PA pressure (mmHg)	22.79 ± 14.89	43.00 ± 16.06	**< 0.0001**
Submassive PE stage (= existing RVD or elivated cTnI levels (>0.1 ng/ml))	10.3 % (6)	100 % (77)	**< 0.0001**

Continuous variables are described by median, 25th and 75th percentile, if they had a skewed distribution (|skewness| >1). Nearly normally distributed variables are presented as mean values and standard deviation. Discrete variables are described through relative and absolute frequencies. Discrete variables were tested with the Chi-square test for contingency tables; continuous variables were analysed with Student’s T Test if they were normally distributed the Mann–Whitney-U test on skewed distribution

Analysis of the ROC curve showed an AUC of 0.79 for cTnI predicting RVD with an optimal cut-off value of 0.01 ng/ml. Of the normotensive PE patients, 58.6 % had cTnI values above 0.01 ng/ml. Sensitivity was 80 % (95 % CI 68–89 %), specificity 67 % (95 % CI 53–80 %), positive predictive value (PPV) 75 % (95 % CI 63–85 %) and negative predictive value (NPV) 73 % (95 % CI 58–85 %). AUC value and cTnI cut-off value predicting RVD as well as sensitivity, specificity, positive and negative predictive value have already been published in another context [24]. We have quoted these results once again to complete the general view on this topic.

**Fig. 2:**
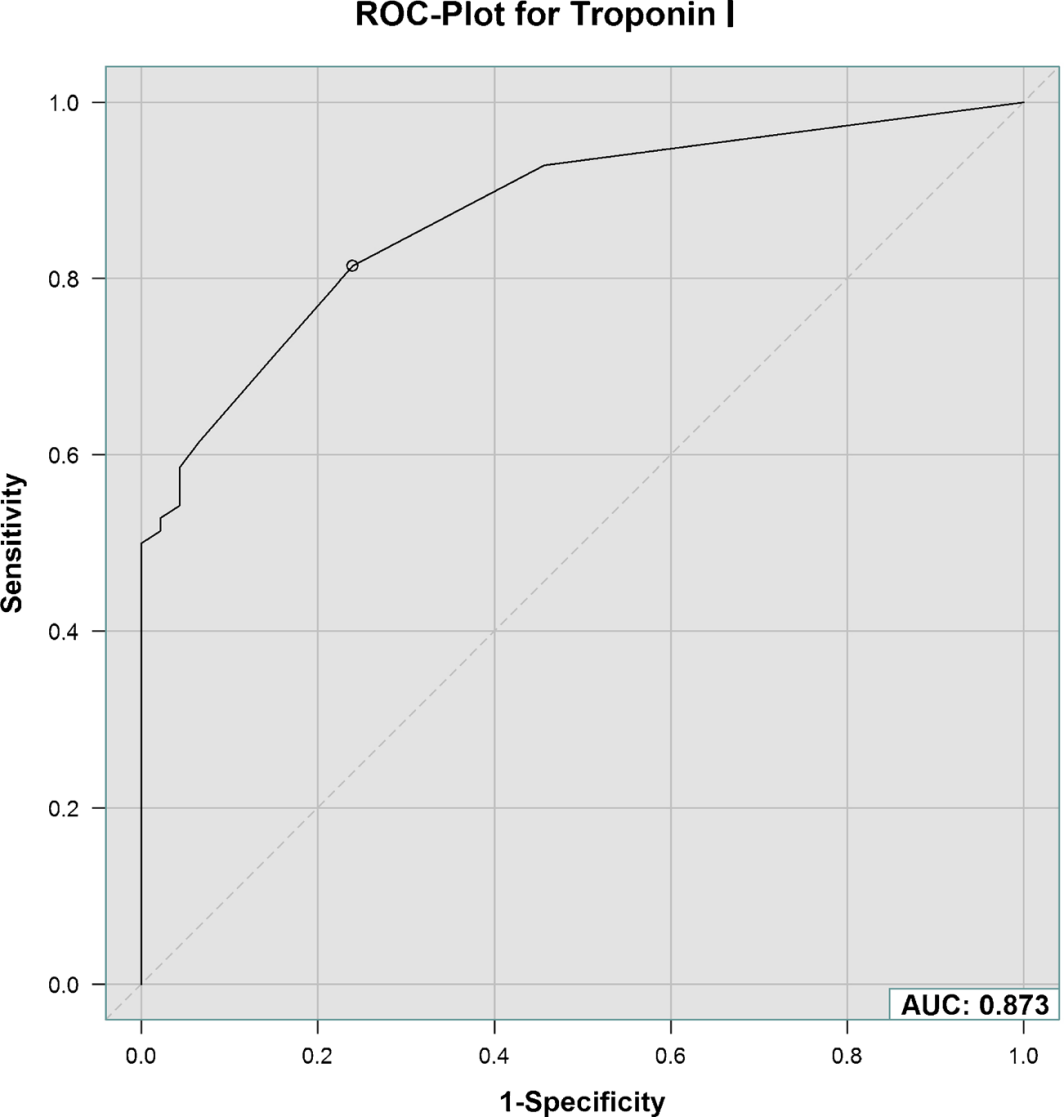
Receiver operating characteristic (ROC) curve with area under the curve (AUC) for effectiveness of cardiac troponin I to predict submassive PE with intermediate risk

Analysis of the ROC curve revealed an AUC of 0.87 for cTnI predicting submassive PE with an optimal cut-off value of 0.01 ng/ml (Fig. 2). Sensitivity was calculated as 81 % (95 % CI 70–90 %), specificity 76 % (95 % CI 61–87 %), PPV 84 % (95 % CI 73–92 %) and NPV 73 % (95 % CI 58–85 %).

**Fig. 3:**
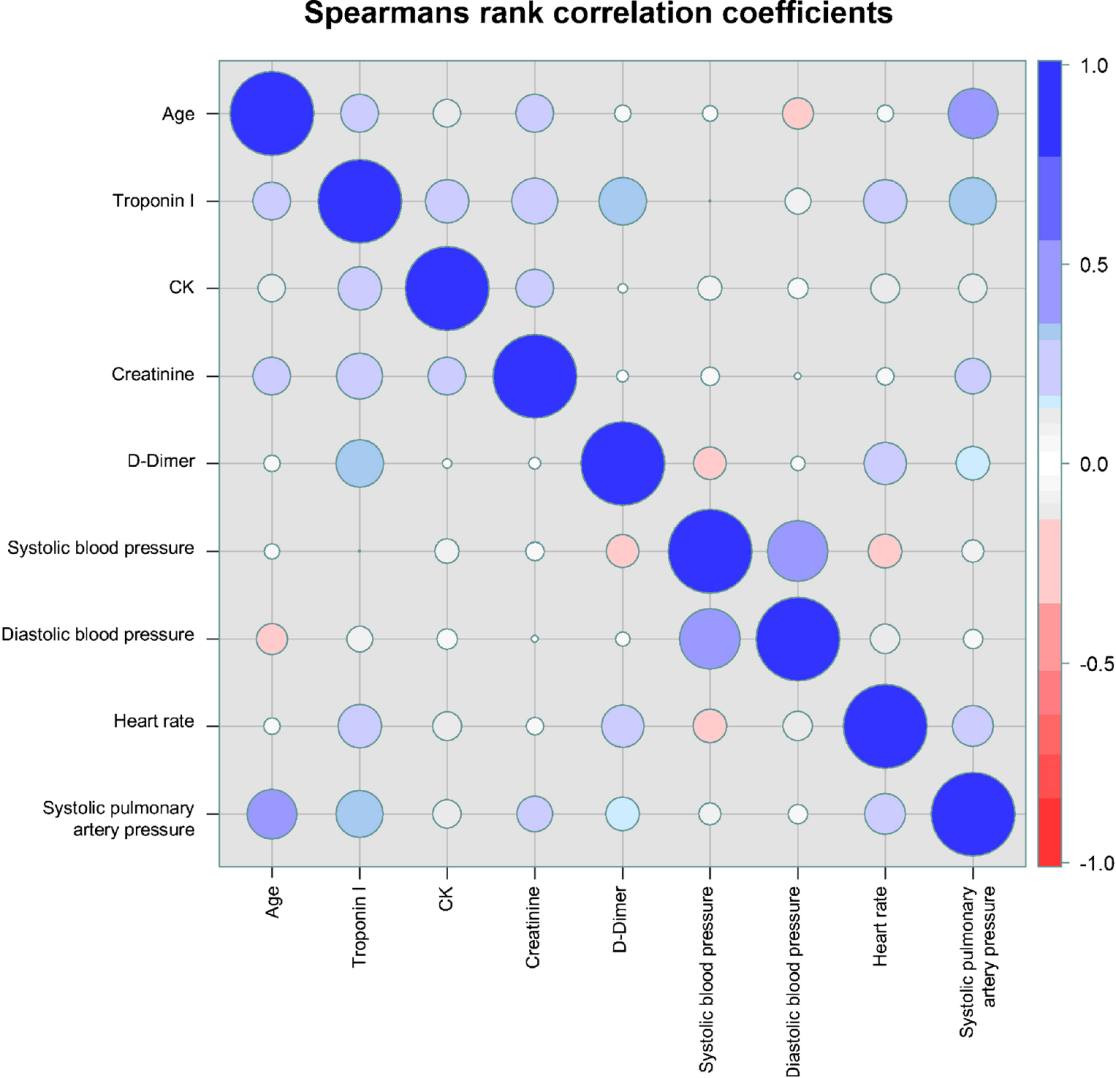
Spearman’s rank correlation coefficient for several parameters

In multivariable logistic regression a strong association between cTnI and RVD was proven (OR 3.95; 95% CI 1.95–8.02, *p* = 0.00014) (Table 2). Spearman’s rank correlation showed a positive correlation between cTnI and D-dimer (*r* = 0.33), sPAP (*r* = 0.32) and creatinine (*r* = 0.31), respectively (Fig. 3).

**Table 2:** Multivariate logistic regression to detect the coherence of RVD and cTnI and other parameters

	OR (95 % CI)	*p*-value
Gender	0.721 (0.263–1.977)	0.52
Age	1.199 (0.715–2.011)	0.49
Log (cardiac troponin I)	3.954 (1.949–8.024)	**0.00014**
CK	1.843 (0.287–11.822)	0.52
Creatinine	1.331 (0.721–2.457)	0.36
D-dimer	0.964 (0.588–1.581)	0.88

## Discussion

Echocardiography, CT and cardiac biomarkers, particularly cTn, are important tools for risk stratification in normotensive PE patients. In acute PE, presence of RVD or elevated cTn levels is associated with increased mortality [1, 2, 4, 7, 9, 10, 12–16, 18–22, 27–31].

Our study confirms a strong association between cTnI and RVD in normotensive PE patients. The calculated OR of 3.95 is in accordance with the literature [27, 32, 33]. AUC for cTnI predicting RVD was 0.79, which is similar to published AUC values of other studies [34, 35].

We calculated a cut-off value of 0.01 ng/ml for cTnI predicting RVD [24], which is in concordance with other study results [34]. Sensitivity, specificity, PPV and NPV were similar to other studies [34] and especially the PPV and NPV were high [24]. These results of AUC, cut-off value, sensitivity, specificity, PPV and NPV were previously published in an investigation for the comparison of three variations of RVD definitions [24]. But, in our opinion, these data are important to complete the general view on this topic and we have therefore quoted them once again.

Only 10.3 % of the PE patients without RVD showed an elevated cTnI value. These patients had myocardial damage without detected RVD and therefore were also categorised as submassive PE with intermediate risk.

At a first glance, calculation of an AUC for cTnI predicting the submassive stage of PE seems to make no sense. But, after careful consideration this calculated AUC could be used to evaluate the importance of the parameter cTnI for assessment of submassive PE. Especially the small difference between the AUC of cTnI predicting RVD and cTnI predicting submassive PE seems to indicate that in our analysis RVD was quite a lot more important for the classification of submassive PE than cTnI. The consistent cut-off values of cTnI for prediction of RVD and submassive PE indicate that the cTnI value to differentiate between normal and pathological is too high. A lower cut-off value for the definition of pathological findings and especially for defining the submassive stage of PE should be chosen in the future.

In a previous investigation, in 179 PE patients of the identified 182 PE patients, we analysed the association of cTnI for risk stratification in haemodynamically unstable and normotensive PE patients together [36]. In the larger analysed group of PE patients, RVD diagnosis was made not only by TTE, but also by CT. In 3 of the 182 patients RVD was not detectable and these 3 patients were therefore not included in the analysis [36]. In these 179 PE patients the association between cTnI and RVD was similar to the present analysis (OR 3.98) [36]. AUC for cTnI predicting RVD was 0.81 with a cut-off level of 0.01 ng/ml [36]. Increasing age, reduced kidney function and higher activation of coagulation system are connected with elevated cTnI values.

In all patients with an acute PE event, anticoagulation therapy is recommended with the aim to prevent early death and to avoid symptomatic recurrent VTE, especially fatal PE [31]. Thrombolytic treatment is not the standard treatment regime in normotensive PE patients, but could be considered if signs of haemodynamic decompensation appear [31]. In the 129 normotensive PE patients of our study we started anticoagulation treatment, but thrombolytic treatment was not given in any of these patients.

## Limitations and strength

Limitations of this study are the small number of included patients and the retrospective character. With these data, we were not able to draw conclusions about follow-up outcome. Despite these limitations we were able to find answers to the main questions of the analysis.

## Conclusions

In normotensive PE patients, cTnI is helpful for risk stratification and especially for exclusion of RVD. Increasing age, reduced kidney function and higher activation of coagulation system are connected with elevated cTnI values.
